# A family of Melnick-Needles syndrome: a case report

**DOI:** 10.1186/s12887-020-02288-2

**Published:** 2020-08-19

**Authors:** Chi Hoon Oh, Chang Ho Lee, So Young Kim, So-Young Lee, Hak Hoon Jun, Soonchul Lee

**Affiliations:** 1 Department of Orthopaedic Surgery, CHA Bundang Medical Center, CHA University School of Medicine, Gyeonggi-do, Republic of Korea; 2 Department of Otorhinolaryngology – Head and Neck Surgery, CHA Bundang Medical Center, CHA University School of Medicine, Gyeonggi-do, Republic of Korea; 3 Department of Internal Medicine, CHA Bundang Medical Center, CHA University School of Medicine, Gyeonggi-do, Republic of Korea; 4 Department of Surgery, CHA Bundang Medical Center, CHA University School of Medicine, Gyeonggi-do, Republic of Korea

**Keywords:** Melnick-Needles syndrome, Osteochondrodysplasia, Family, *FLNA*

## Abstract

**Background:**

Melnick-Needles syndrome (MNS) is an extremely rare osteochondrodysplasia caused by a mutation of *FLNA,* the gene encoding filamin A. MNS is inherited in an X-linked dominant manner. In this study, we describe three members of the same family with MNS, who exhibited different phenotypic severity despite having an identical *FLNA* gene mutation.

**Case presentation:**

The patient was 16 months old, with a history of delayed physical development, multiple upper respiratory infections and otitis media episodes. She was referred to our orthopedic clinic because of bowed legs and an abnormal plain chest radiograph. Both upper and lower extremities were bowed. Plain X-rays showed thoracolumbar kyphoscoliosis, with anterior and posterior vertebral scalloping, and thin, wavy ribs. Hypoplasia of the pubis and ischium, with bilateral coxa valga, were also noted. Target exome sequencing revealed a heterozygous mutation of *FLNA*, c.3578 T > C, p.Lys1193Pro, which confirmed the diagnosis of MNS. Her older sister and mother had minimal deformities of the axial and extremity skeleton, but genetic analyses revealed the same *FLNA* mutation as the patient. The mutation identified in this family has not been previously reported.

**Conclusion:**

This report illustrates the potential inherited nature of MNS and the phenotypic variability of clinicoradiologic characteristics. In patients with traits suggestive of MNS, a careful medical and family history should be obtained, and genetic testing should be performed for the patient, as well as all family members.

## Background

Melnick-Needles syndrome (MNS, OMIM: #309350) is an extremely rare osteochondrodysplasia [1–3]. To date, less than 70 cases of MNS have been reported worldwide [4]. MNS is caused by gain-of-function mutations in the *FLNA* gene (OMIM: #30017) which encodes filamin A. Patients with MNS typically have unusual facial features, short ribbon-like ribs, scoliosis, bowing of the long bones, and vertebral scalloping [5]. Intelligence is not impaired. In more severe cases, affected individuals die in the second or third decade of life from respiratory failure secondary to the chest wall abnormalities [4].

MNS is a member of a group of five X-linked diseases with overlapping clinical phenotypes, known collectively as otopalatodigital syndrome (OPS) spectrum disorders [6]. Other members of the group are OPS type 1 (OMIM: #311300), OPS type 2 (OMIM: #304120), frontometaphyseal dysplasia (FMD, OMIM: #305620), and terminal osseous dysplasia with/without pigmentary defects (TODPD, OMIM: #300244). MNS is found almost exclusively in females, as the syndrome is lethal during gestation or the perinatal period in almost all affected males [7]. In males that do survive to term, the phenotype is clinically indistinguishable from that of OPS type 2 [8]. Females with MNS have characteristic clinical and radiologic diagnostic findings. Table 1 shows details of the clinical features of patients with MNS reported in the past 15 years [9–14].

**Table 1:** Summary of Melnick-Needles syndrome case reports within the past 15 years

Case	Year	Sex	Age	Described clinical features
1 [13]^a^	2017	F	27	Mandibular hypoplasia, retrognathia sleep apnea
2 [13]^a^	2017	F	21	Mandibular hypoplasia, retrognathia, hypodontia
3 [12]	2016	F	13	Cranial hyperostosis, short upper limbs, bowed long bones, metaphyseal thickening, genu valgum, shortened distal phalanges, hypoplastic pelvis and shoulders, rib tapering and irregularities, elongation of vertebrae, kyphoscoliosis, micrognathia, mandibular hypoplasia, abnormal dental development
4 [10]	2013	F	17	Prominent forehead, severely deformed chest with a significant mid-thoracic kyphosis, genu valgum, limb length inequality
5 [10]	2013	F	18	Prominent eyes, full cheeks, small chin, large prominent forehead, genu valgum, low weight and small height, significant lung disease (stent in right main bronchus)
6 [14]	2012	F	18	Unfavourable aesthetics, masticatory problems, sigmatism in her speech, sclerosis of the skull base, moderate kyphoscoliosis, curved clavicle, small rib cage, lowed long bones with metaphyseal flaring, coxa valga, hypoplastic pelvis
7 [9]	2009	F	6	Exophthalmos, full cheeks, high forehead, micrognathia, malaligned teeth, genu valgum, small chest wall with pectus carinatus, low weight and small height
8 [11]	2006	F	39	Dyspnea with congestion and wheezing, micrognathia, small and crowded oropharynx, kyphoscoliosis

^a^Cases 1 and 2 are sisters. In other cases, however, there is no familial report, so there is no information about inheritance

Because MNS is extremely rare and some cases are lethal, mostly it is detected as de novo condition, but once it is developed, it is inherited in an X-linked dominant manner [15, 16]. However, previous reports did not describe the familial characteristics of the disease in detail. In this case report, we describe a family with MNS who exhibited variable severity of phenotypic changes despite having an identical *FLNA* gene mutation.

## Case presentation

**Fig. 1:**
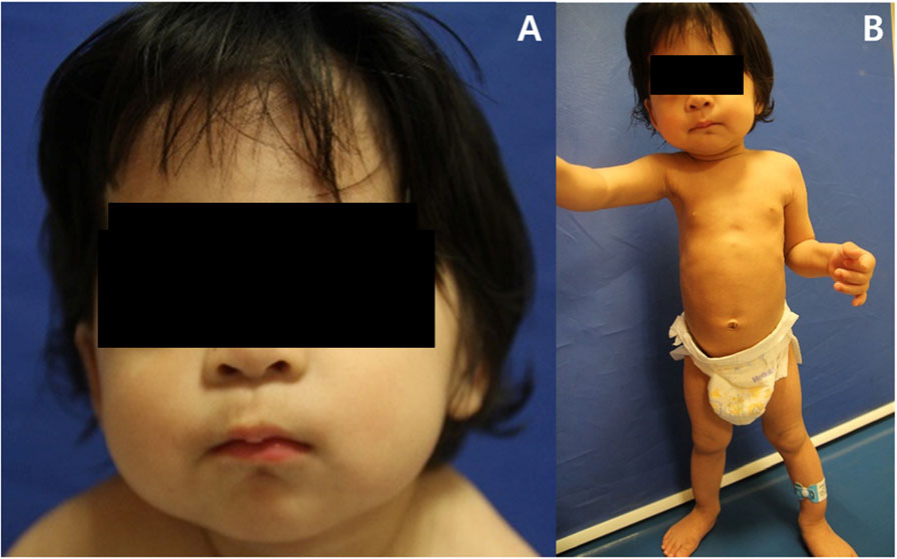
Photographs showing general morphology of the patient at age 16 months. **a** The patient’s face exhibited prominent eyes, supraorbital hyperostosis, full cheeks, and micrognathia. **b** Her legs and arms were thin and curved

A 16-month-old female was referred to our orthopedic department because of bowed legs and an abnormal chest X-ray. She was 80 cm (50th percentile) tall and weighed 9.7 kg (25th percentile). Her past medical history was positive for delayed physical development, as well as recurrent respiratory tract infections and episodes of otitis media. Examination revealed several facial characteristics of MNS, including prominent eyes, supraorbital hyperostosis, full cheeks, and micrognathia (Fig. 1). Plain radiographs revealed a number of abnormalities.

**Fig. 2:**
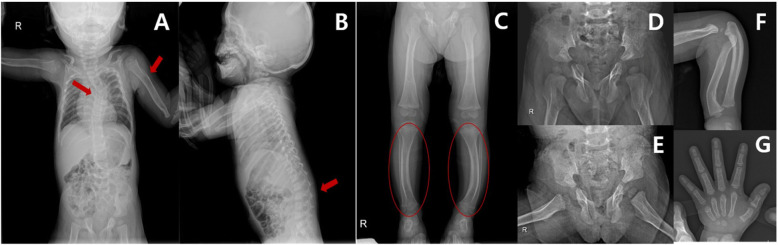
Plain radiographs of the patient at age 16 months. Overall, the patient’s bones were curved and thin. Bone age was not delayed, but her bones did not have normal alignment or cortical bone maturity and showed osteodysplasia. **a**, **b**, red arrow The patient had a thoracolumbar kyphoscoliosis with a humeral cortical irregularity and thin, wavy ribs. **c**, red circle Bilateral bowed leg deformities with Erlenmeyer flask deformity were observed. **d**, **e** The pubis and ischium were hypoplastic, and coxa valga was present bilaterally. **f** The ulna and radius were curved. **g** Bone age was normal, according to the hand radiographs

X-ray showed thoracolumbar kyphoscoliosis and anterior and posterior vertebral scalloping; humerus cortical irregularity with bowing; thin wavy ribs; pelvis hypoplasia of the pubis and ischium and bilateral coxa valga; bilateral bowed leg deformities, with normal epiphyses and metaphyses. The bone age estimated from X-rays of the hand was 1.5 years, which was similar with the patient’s chronological age (Fig. 2).

To confirm the clinical impression of MNS, we performed genetic analyses. Target exome sequencing revealed a heterozygous mutation in the *FLNA* gene, c.3578 T > C, p.L1193P, which confirmed the diagnosis. Subsequently, the patient continued to have repeated respiratory tract infections and otitis media episodes. She died at the age of 6 years of a cardiac arrest, the direct cause of which was undetermined.

**Fig. 3:**
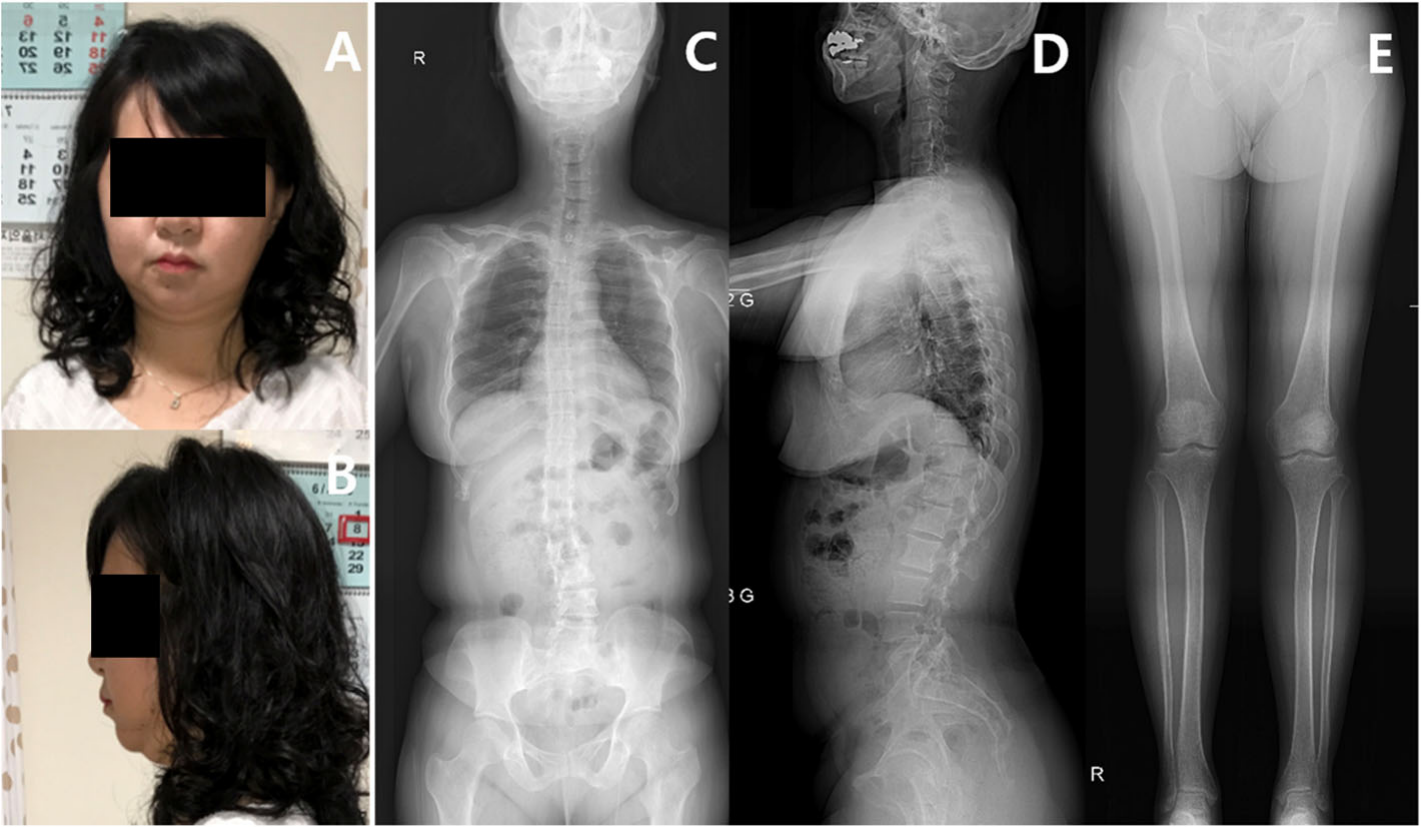
Photographs and plain radiographs of the patient’s mother. Overall, the patient’s mother had relatively mild deformities, when compared with the patient. **a**, **b** Like the patient, she had full cheeks and micrognathia,which were mild. **c**, **d**, **e** She also had mild lumbar scoliosis, kyphosis, and minimally bowed legs bilaterally

**Fig. 4:**
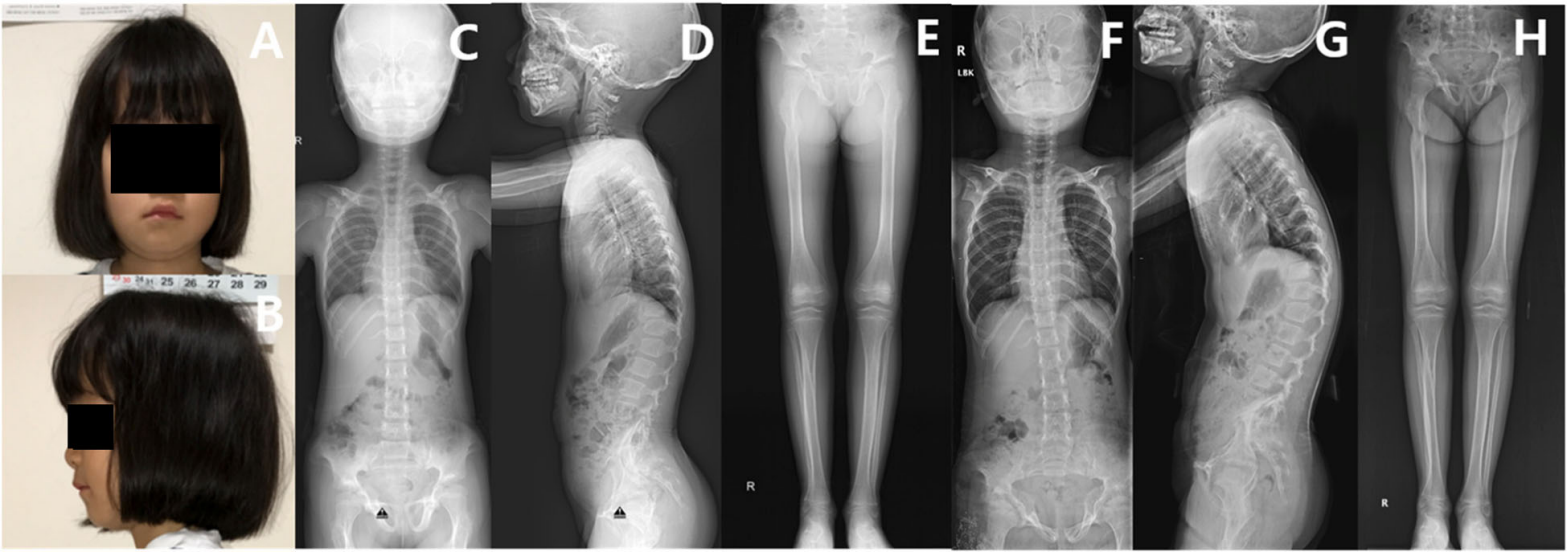
Photographs and plain radiographs of the patient’s older sister. **a**, **b** The patient’s older sister had a nearly normal-appearing face. **c**, **d** At 6 years of age, plain radiographs revealed normal spine alignment. **e** However, bilateral coxa valga and Erlenmeyer flask deformities were noted at 6 years of age. **f**, **g** At age 9 years, thoracolumbar kyphoscoliosis was apparent. **h** The coxa valga and Erlenmeyer flask deformities were still present

Target exome sequencing was also performed for all available family members. Her mother and older sister were found to have the exact same mutation, although they exhibited less severe MNS phenotypes. Her mother had full cheeks (like the patient) and lumbar scoliosis, but she had minimal leg deformities (Fig. 3). Her older sister had essentially no MNS facial characteristics, but she did exhibit coxa valga deformities (Fig. 4). When initially assessed, the sister’s spinal alignment was normal, but thoracolumbar kyphoscoliosis was observed at 9 years of age.

## Discussion and conclusions

The locus associated with MNS is the *FLNA* gene, which encodes the cytoskeletal protein filamin A. *FLNA* comprises 48 exons and encodes a modular protein with an N-terminal actin-binding domain and a tail of 24 structurally homologous repeats [4]. Cellular functions mediated by filamin include linking signal transduction events to modulation of the actin cytoskeleton and gene transcription [17]. In 2003, Robertson et al. reported that MNS is caused by gain-of-function mutations in the *FLNA* gene and has an X-linked pattern of inheritance. They also noted that *FLNA* mutations are responsible for OPS type 1, OPS type 2, FMD, and TODPD (Table 2) [6, 12, 18].

**Table 2:** Comparisons of key features of OPS spectrum disorders [6, 12, 18]

Type	Sex^a^	Prognosis	Skeletal dysplasia	Craniofacial anomaly	Other features
**MNS**	Male	Similar with OPD type 2 but more severe manifestation, dies during embryonic period.			
	Female	1.Substantial variability is observed in females. 2. Normal fertility 3. Normal intelligence	1. Flexed upper limbs 2. Postaxial polydactyly 3. Bowed limb 4. Clubfeet 5. Kyphoscoliosis 6. Short stature 7. Thoracic hypoplasia 8. Joint subluxation	1. Large fontanelles 2. Malar flattening 3. Bilateral cleft palate 4. Bifid tongue 5. Severe micrognathia 6. Prominent supraorbital ridges 7. Proptosis 8. Full cheeks	1. Fibrosis of pancreas and spleen 2. Bilateral cystic renal dysplasia 2ndary to obstructive uropathy and omphalocele 3. Oligohypodontia 4. Hearing loss (Common) 5. Hydronephrosis 2ndary to ureteric obstruction (Common) 6. Bleeding diathesis
**OPS type 1**	Male	1. Phenotypes are evident at birth. 2. No late-onset orthopedic complications 3. Normal life span 4. Normal fertility 5. Normal intelligence	1. Hypoplasia of thumbs, distal phalanges, great toe, a long second toe 4. Joint contracture (Wrist, elbow) 5. Bowed limb (Mild) 6. Reduced stature (Mild)	1. Supraorbital hyperostosis 2. Downslanted palpebral fissures 3. Widely spaced eyes 4. Wide nasal bridge and broad nasal tip	1. Hearing loss 2. Cleft palate
	Female	Variable clinical severity			
**OPS type 2**	Male	1. Neonatal lethality due to usually from thoracic hypoplasia resulting in pulmonary insufficiency 2. Developmental delay	1. Thoracic hypoplasia 2. Bowed limb 3. Short stature 4. Hypoplasia of thumb & big toe 5. Delayed closure of fontanelles 6. Scoliosis	Similar with male of OPD type 1 but more severe manifestation	1. Hearing loss 2. Cardiac septal defects 3. Omphalocele 4. Hydronephrosis 2ndary to ureteric obstruction 5. Hypospadias 6. Hydrocephalus, cerebellar hypoplasia
	Female	Usually present with a subclinical phenotype			
**FMD**	Male	Normal intelligence	1. Hypoplasia of distal phalanges 2. Progressive joint contractures (Hand IP & MP, wrist, elbow, knee, ankle) 3. Progressive scoliosis 4. Bowed limb	1. Very pronounced supraorbital hyperostosis 2. Downslanted palpebral fissures 3. Widely spaced eyes	1. Hearing loss 2. Oligohypodontia (Frequent) 3. Underdevelopment of the muscle around the shoulder girdle & in the intrinsic muscles of the hands (Common) 4. Subglottic stenosis 5. Urethral stenosis, and hydronephrosis
	Female	Characteristic craniofacial features similar to those of affected males			
**TODPD**	Male	A male presentation of TODPD has never been described.			
	Female	Normal intelligence	1. Disorganized ossification of the carpals and metacarpals. 2. Marked camptodactyly 3. Bowed limbs 4. Radial head dislocation 5. Short stature 6. Scoliosis.	1. Widely spaced eyes 2. Punched out hyperpigmented lesions characteristically over the temporal region. (Unlike the fibromata, these lesions do not involute with age.)	1. Digital fibromata appear in infancy, eventually involute before age ten years. 2. Cardiac septal defects 3. Ureteric obstruction (Occasional) 4. Alopecia (Variable)

^a^In general, female patient shows mild phenotype compared to male

*OPS* Otopalatodigital syndrome, *MNS* Melnick-Needles syndrome, *FMD* Frontometaphyseal dysplasia, *TODPD* Terminal osseous dysplasia with pigmentary skin defects, *IP* Interphalangeal, *MP* Metacarpophalangeal

The pathogenesis of MNS has not been established. Some researchers have reported increased skeletal collagen content, which could explain the sclerosing bone process [9]. Fryns et al. suggested that MNS was a generalized connective tissue disorder because of the hyperlaxity of skin and joints [19]. Urological, pulmonary, and cardiac involvement is also common in patients with MNS [20, 21]. Although diverse phenotypes may occur [22], affected females are usually short and may have delayed motor development, osteoarthritis, a hoarse voice, and urethral stenosis (leading to hydronephrosis), in addition to the main abnormalities [1].

It is not yet known why phenotypes may differ between females with MNS. Skewed X-inactivation and somatic mutation have been suggested as potential mechanisms [23, 24]. For example, Robertson et al. reported monozygotic twin sisters, only one of whom had MNS [24]. In our case, although phenotypic severity differed between female family members, they all had the same heterozygous *FLNA* mutation (c.3578 T > C, p.L1193P). Interestingly, the mutations identified in this family have not been previously reported in the National Center for Biotechnology Information’s ClinVar. As mentioned earlier, pathogenesis of MNS has not been established well. Further research is also needed to determine pathogenicity of this mutation among these families with MNS.

It should be noted that the patient with MNS would demonstrate the typical bone deformity in the distal femur, which was called as the Erlenmeyer flask deformity like Camurati-Engelmann disease (CED, OMIM: #131300). CED is another rare genetic skeletal disorder caused by tumor growth factor-β1 mutation, which is characterized by limb pain, muscle emaciation and weakness, cortical thickening of the diaphysis of long bones, and also Erlenmeyer flask deformity [25, 26].

Although MNS is rare, physicians should be aware of the disorder, including its variable manifestations, because of the potential lethality of severe disease. A possible hereditary mutation should be suspected even when family members have an almost normal appearance. Thus, in patients exhibiting traits suggestive of MNS, imaging studies and genetic testing should be performed for both the patient and all family members.

### Informed consent statement

Informed written consent was obtained. It contains publication of this report and the accompanying images, including photographic rights of patient, her mother and her sister (Use of medical information for academic purposes, including the portrait rights shown in Figs. 1, 3 and 4). For the patient, her mother and her sister, written consent was obtained from the father and mother for all teaching and academic purposes, including publication of this case report.

## Data Availability

The datasets used and/or analyzed during the current report are available from the corresponding author on reasonable request.

## Abbreviations

MNS: Melnick-Needles syndrome
OPS: Otopalatodigital syndrome
FMD: Frontometaphyseal dysplasia
TODPD: Terminal osseous dysplasia with/without pigmentary defects
CED: Camurati-Engelmann disease
